# Understanding airflow dynamics: a computational study of nasal and oral breathers using patient-specific models

**DOI:** 10.1038/s41405-025-00357-1

**Published:** 2026-01-15

**Authors:** Shawn Ann Thomas, Supriya Nambiar, Mohammed Zuber, S M Abdul Khader, Athira V

**Affiliations:** 1 Department of Orthodontics and Dentofacial Orthopaedics, Manipal College of Dental Sciences Mangalore, Manipal Academy of Higher Education, Manipal, Karnataka 576104 India; 2 Manipal Institute of Technology, Manipal Academy of Higher Education, Manipal, Karnataka 576104 India

**Keywords:** Craniofacial orthodontics, Cone-beam computed tomography

## Abstract

**Objective:**

Breathing patterns and their influence on craniofacial growth and development have remained a subject of debate within orthodontics and otolaryngology. In this study, Computational Fluid Dynamics (CFD) was employed to analyze and compare airway morphology and airflow dynamics between individuals with normal nasal breathing and those with varying degrees of nasal obstruction.

**Materials and methods:**

A total of five patients aged 18 to 28 years were selected for the study, comprising four individuals with varying degrees of nasal obstruction and one asymptomatic nasal breather, as verified by prior clinical evaluation from an otorhinolaryngologist. The samples were categorized into three groups: (1) nasal breathers with no anatomical abnormalities, (2) mouth breathers with septal deviation, and (3) predominant mouth breathers with nasal polyposis. High-resolution computed tomography (CT) scans were utilized for airway segmentation and subsequent CFD analysis. Key parameters like airflow patterns, velocity distribution, WSS, and airway resistance were evaluated at multiple sites within the pharyngeal airway.

**Results:**

The analysis revealed a non-uniform velocity distribution within the pharyngeal airway, influenced by anatomical variations in the nasal cavity. Flow parameters, including velocity streamlines, velocity contours, WSS, and pressure drop, varied notably across different grades of nasal obstruction.

**Conclusion:**

The observed airflow characteristics offer valuable insights into the combined effects of physiological and pathological breathing on airway dynamics. The airflow patterns identified in this study confirm the presence of altered airflow in obstructed nasal airways. Flow parameters, such as velocity streamlines, which illustrate the trajectory of air through the nasal cavity, may serve as supportive indicators for clinical symptoms such as anosmia. The non-invasive nature of CFD allows for a realistic assessment of airway flow behaviour.

## Introduction

Breathing patterns and their influence on craniofacial growth and development have long been a subject of continued investigation and debate within the fields of orthodontics and otolaryngology [1]. Continuous airflow through the nasal passages during breathing provides a consistent stimulus that supports the growth and development of the craniofacial complex. Impaired nasal breathing, often caused by conditions such as allergic rhinitis or nasal trauma, remains a significant concern [2]. It has been reported in literature that 1–4% of patients with nasal polyposis exhibit impaired nasal breathing [3]. Studies investigating airway size and shape have traditionally relied on lateral cephalometric radiographs as a measurement tool. However, concerns have been raised regarding the validity of this method, as lateral head films provide only a two-dimensional representation of a complex three-dimensional airway structure. Variations in head posture have been shown to influence airway measurements within the same individual, indicating that airway dimensions are posture-dependent. Capturing the complex behavior of airflow within the human airway remains challenging when relying solely on invasive experimental methods and clinical assessments.

Advancements in radiographic imaging have greatly enhanced the evaluation of airway structures in both orthodontics and respiratory health. 3D imaging enables the application of Computational Fluid Dynamics (CFD) to evaluate pharyngeal airflow by analyzing airflow characteristics from multiple perspectives. CFD offers a non-invasive approach to analyzing airflow patterns, particularly in complex airway structures, influencing nasal resistance and function. It accurately evaluates airflow by incorporating patient-specific airway geometry into the analysis. This technique numerically governs fluid movement and separately evaluates airflow within the nasal cavity, offering a more accurate regional assessment. Chen et al. [4] conducted a CFD-based study to investigate pharyngeal vibrations and pressure fluctuations caused by uvula flapping in pediatric airway models, while Zheng et al. [5] examined the alterations in the upper airway following significant anterior retraction in Class I bimaxillary protrusion patients.

Airway assessment is an important part of orthodontic examination, as studies have shown that increased airway resistance can lead to abnormal development of the nasomaxillary complex. To the best of our knowledge, airway characteristics in individuals with increased airway resistance have not yet been thoroughly investigated. This study aims to evaluate airway dynamics in patients with anatomical nasal obstructions by comparing normal and obstructed airways using 3D models derived from computed tomographic images. Additionally, parameters such as airway volume, velocity, wall shear stress (WSS), and pressure distribution were analyzed to establish objective criteria for distinguishing between obstructive and anatomical oral breathers.

## Materials and methodology

The present observational study was conducted following approval from the Institutional Ethics Committee (protocol no: 18133). CT images of the selected patients were retrieved from the teaching hospital archives to create virtual three-dimensional airway models.

The study included five adult patients: four with nasal obstruction (two males and two females) and one female patient with no symptoms of nasal obstruction, as confirmed by an otolaryngologist. The patient cohort consisted of normal nasal breathers, mouth breathers with septal deviation, and individuals with nasal polyposis. The samples were categorized into three groups: (1) nasal breathers with no anatomical abnormalities, (2) mouth breathers with septal deviation, and (3) predominant mouth breathers with nasal polyposis. High-resolution CT scans of adults aged 18–28 years with a Body Mass Index (BMI) of 25.2 ± 4.2 kg/m² were analyzed.

Inclusion criteria comprised individuals with a history of nasal breathing difficulties, no prior nasal surgery or medication use, and no pulmonary or cardiovascular conditions affecting breathing patterns. Exclusion criteria included a history of maxillofacial trauma, nasal bone fractures, respiratory illnesses, systemic autoimmune diseases, and prior orthodontic or orthopedic treatment.

The recorded case history and clinical examination of the patients performed by an otolaryngologist were reviewed to confirm the breathing pattern of the individuals. In normal breathers, CT scans showed patent pharyngeal airways without septal deviation or nasal polyps. Patients with nasal obstruction exhibited nasal septum deviation, hypertrophy of the middle and inferior turbinates on anterior rhinoscopy, mild to moderate exertional dyspnea, and nasal resistance exceeding 0.50 Pa m^3^/s.

All participants provided informed consent prior to their inclusion in the study. They were fully informed about the nature, purpose, and procedures of the research. Participants consented to participate voluntarily and agreed to the use of their anonymized data for research and publication purposes.

### Three-dimensional reconstruction of patient-specific upper airway models

High-resolution CT images of six patients were utilized to generate precise, patient-specific 3D models. Scans were conducted from the lower rim of the epiglottis to the supraorbital margin, with a spatial resolution of 512 × 512 pixels, zero spacing, and a slice thickness of 1.25 mm, producing images in Digital Imaging and Communication in Medicine (DICOM) format. Scanning was performed while patients were awake, positioned supine and neutral, during a single breath-hold at the end of normal inspiration, without swallowing, and in centric occlusion. The acquired DICOM images were then imported into commercial software (Mimics 13.0, Materialise, Belgium) for airway segmentation, using a threshold value defined in Hounsfield Units.

**Fig. 1:**
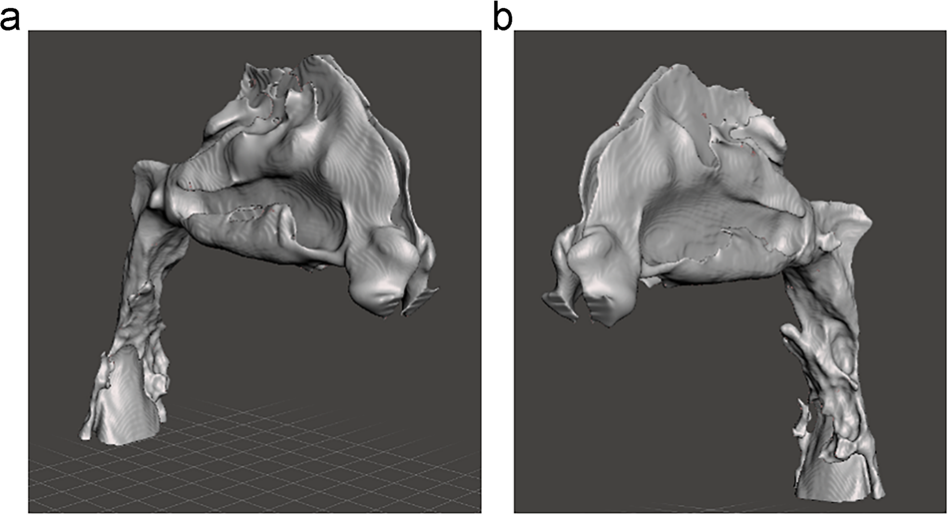
The three-dimensional segmented models including the nostrils, the nasal cavity, the nasopharynx, the velopharynx, and the oropharynx and ending below the epiglottis. **a** Three-dimensional patient specific airway models of right side. **b** Three-dimensional patient specific airway models of left side.

The three-dimensional segmented models encompassed the nostrils, nasal cavity, nasopharynx, velopharynx, and oropharynx, extending down to the epiglottis (Fig. 1a, b). Segmentation was performed on a slice-by-slice basis, with manual refinement applied to correct leakage in the segmentation mask and eliminate unwanted regions erroneously connected to the upper airway model.

### Mesh generation

The finalized 3D airway model was initially exported in STL format and imported into CATIA, where the Digitized Shape Editor module was employed to identify and correct surface irregularities, such as holes and distortions. This process was further refined using Quick Surface Reconstruction software to ensure geometric continuity and accuracy. The model then underwent additional processing in 3-Matic (Materialise, Belgium), where smoothing and surface meshing were performed to improve the fidelity of the anatomical representation. This step also enabled the precise demarcation of critical anatomical features, including the airflow inlet (nostrils), outlet (epiglottic base), and airway walls. Mesh generation played a key role in enhancing model quality by re-meshing and smoothing the geometry, ensuring the structure was optimized for computational stability and accuracy. Volume meshes were then generated from the surface mesh using ANSYS Fluent Meshing (Version 19, ANSYS, Canonsburg, USA). High-resolution, unstructured grids were created, tailored to the complex anatomy of the upper airway, with the final models consisting of approximately 4.4 × 10^5^ to 1.5 × 10^6^ cells, depending on anatomical variation. Finally, the prepared geometry was exported in .stp format and imported into ANSYS FLUENT for numerical simulation of airflow dynamics.

### Boundary conditions

**Fig. 2:**
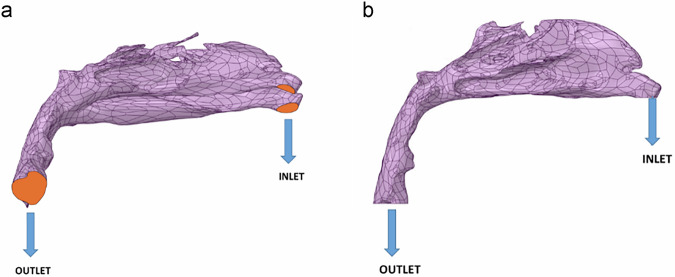
Three-dimensional patient-specific airway models with the nostril as the inlet and the base of the epiglottis as the outlet. **a** Three-dimensional patient-specific airway models with nostril as the inlet and base of the epiglottis as the outlet (right side). **b** Three-dimensional patient-specific airway models with nostril as the inlet and base of the epiglottis as the outlet (left side).

ANSYS FLUENT (USA) was used to simulate airflow within patient-specific airway models, with the nostrils defined as the inlet and the base of the epiglottis as the outlet (Fig. 2a, b). The simulation was performed under steady-state conditions using a constant flow rate of 0.0002 kg/s, with corresponding inlet velocities derived from this rate. A no-slip boundary condition was applied to the airway walls to provide a realistic approximation of airflow behavior. To simulate inspiration, a mass flow inlet boundary condition was imposed at the nostrils, corresponding to a flow rate of approximately 14 L/min, reflective of normal breathing conditions.

### Governing equations

In this study, air was treated as a Newtonian fluid, meaning its flow behavior generates viscous stresses that are linearly proportional to the local strain rate over time. As a result, the stresses at any given point are directly related to the rate of change in the fluid’s velocity vector. The equations for assessing the flow properties are the continuity and Navier-Stokes equations.

Continuity equation: $$\nabla \cdot u=0$$

Components of velocity vector density: $u\cdot \nabla u=-\frac{1}{\rho }\nabla {p}+{v}{\nabla }^{2}u$

where symbols *u*, *ρ*, and *p* represent the velocity of air, the fluid’s density and pressure, respectively, while ‘*v*’ denotes the fluid’s kinematic velocity.

In the simulations, the density of air was considered as 1.225 kg m^−3^. Reynold’s numbers at the inlet and outlet were 1836 and 1614 for all the models. The computational analysis would yield a pressure drop (Δ*P*) for the flow rates (Q) studied. The airway resistance could be calculated from these values by using the equation, $R=\Delta P/Q$.

### Solution methodology

After transforming the governing equations of fluid flow (Navier–Stokes and continuity equations) into their integral forms, they were numerically integrated over the computational mesh representing the airway geometry. This discretization process yielded a system of algebraic equations, which were solved to compute pressure and velocity fields using ANSYS FLUENT 2024 *R*2.

Throughout the iterative solution process, residuals were continuously monitored to evaluate numerical errors and ensure solution stability. Convergence was assessed based on the reduction of these residuals, with the standard criterion set to a minimum of four orders of magnitude. Achieving this level of convergence was essential to ensure the reliability and accuracy of the simulation results.

## Results

### Velocity streamline characteristics

**Fig. 3:**
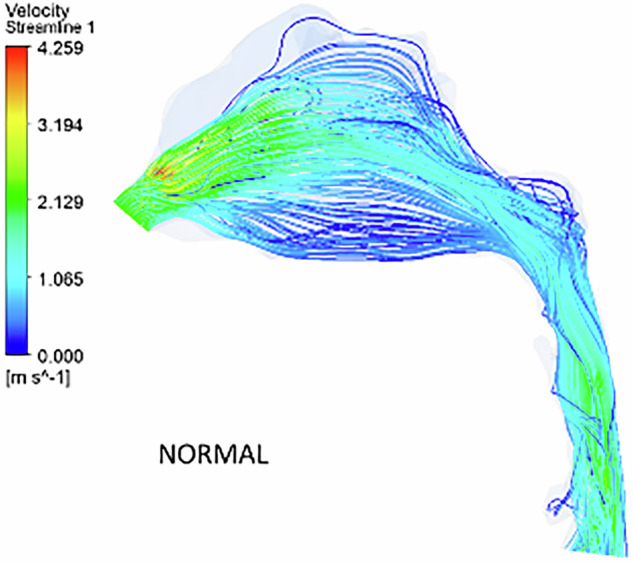
The velocity streamlines in a healthy nose model.

According to Fig. 3, in the healthy nasal model, airflow within the pharyngeal airway primarily moves through the middle and floor of the nasal cavity, as depicted by velocity streamlines. Minimal vortex formation is observed throughout the region. While a decreasing airway tendency is noted from the nostril to the nasal valve region, it is less pronounced compared to other models. The airflow velocity increases at the nasal valve region, reaching 4.3 m s^−1^, with the highest velocity occurring between the posterior end of the nasal valve and the anterior end of the turbinates.

**Fig. 4:**
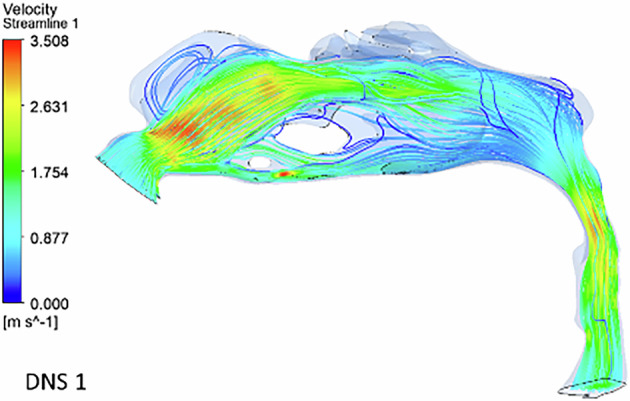
The velocity streamlines in a deviated nasal septum I.

In the deviated nasal septum model, airflow distribution within the pharyngeal airway is altered (Fig. 4). The airflow bypasses obstructed regions in the middle meatal area, redirecting towards normal regions of the nasal cavity. A velocity spike is observed in the superior and inferior meatus regions, with most airflow concentrated in the superior turbinate region. The olfactory region shows an absence of airflow streamlines. A gradual reduction in airflow is observed from the nostril to the nasal valve region. The inlet velocity at the nostrils is 1 m s^−1^, but as it reaches the constricted nasal valve region, it spikes to 3.5 m s^−1^. The minimum cross-sectional area also exhibits increased velocity (3.2 m s^−1^), with airflow streamlines densely packed in a narrow region.

**Fig. 5:**
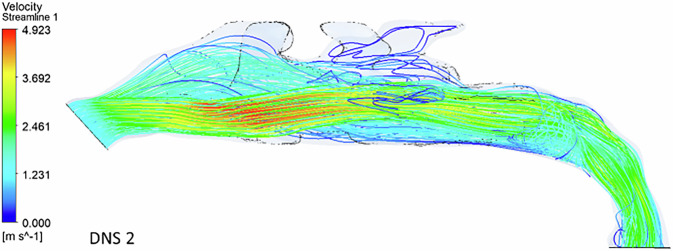
The velocity streamlines in a deviated nasal septum II.

In the second deviated nasal septum model, airflow is predominantly redirected to the inferior turbinate region, leading to reduced flow through the superior turbinate region (Fig. 5). Higher velocity airflow is observed behind the nasal valve in the inferior turbinate region. Unlike the first deviated septum model, there is no sudden spike in velocity between the nostrils and the nasal valve region. The flow dynamics indicate an abnormal pattern, with major airflow passing through the middle meatal region and little to no flow through the superior turbinate region. Minimal airflow is observed in the inferior turbinate region.

**Fig. 6:**
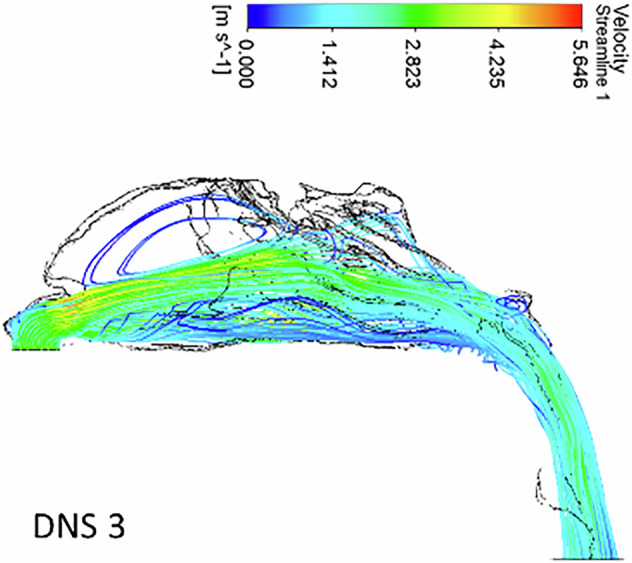
The velocity streamlines in a deviated nasal septum III.

In the third deviated nasal septum model (Fig. 6), the inflow velocity at the nostrils is 1 m s^−1^, but a significant spike to 4.2 m s^−1^ occurs at the nasal valve region.

**Fig. 7:**
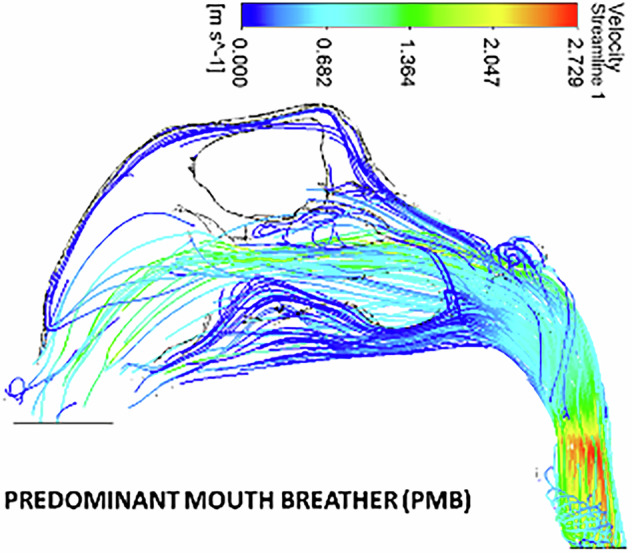
The velocity streamlines in a unilateral grade III polyp.

**Fig. 8:**
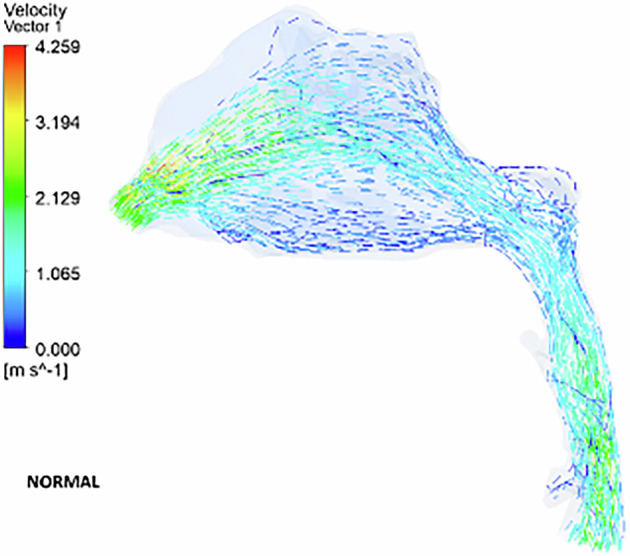
The airflow velocity vectors in a healthy nose model.

In the polyposis model, airflow is redirected locally around obstructions in the superior turbinate region. The middle turbinate region exhibits a higher flow velocity (0.8 ms^−1^) compared to the inferior turbinates (0.5 m s^−1^). The inlet velocity at the nostrils remains constant throughout the nasopharynx, with no significant velocity change in the nasal valve region. The velocity at the nasal valve region measures 0.8 ms^−1^. The minimum cross-sectional area demonstrates an increased velocity of 2.7 ms^−1^, with flow streamlines concentrated in a narrow passage (Figs. 7, 8)

### Velocity vector

**Fig. 9:**
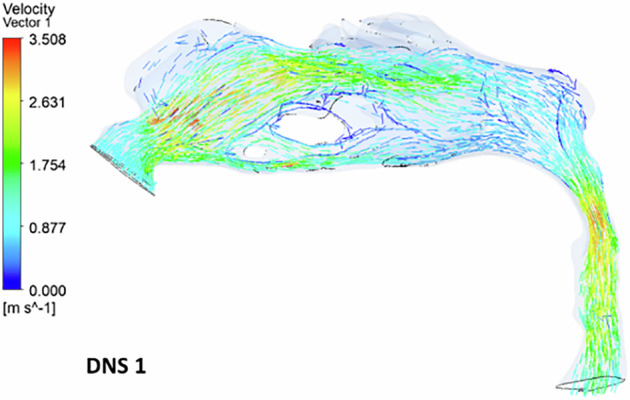
The airflow velocity vectors in a deviated nasal septum I.

**Fig. 10:**
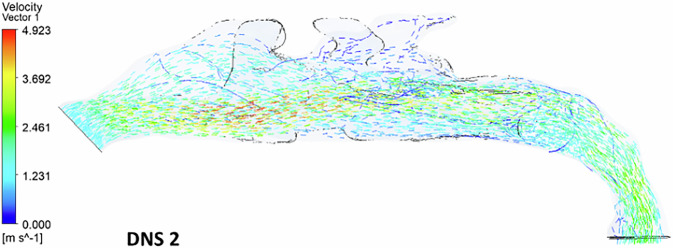
The airflow velocity vectors in a deviated nasal septum II.

The velocity vector illustrates the airflow direction through the pharyngeal region. In the deviated nasal septum model, higher airflow velocities (3.2–3.5 ms^−1^) are concentrated in the middle meatal region, while the superior and inferior meatal regions exhibit lower velocities. Areas of airflow re-circulation are observed in the olfactory region, particularly in the superior nasal conchae. Additionally, a section of the middle meatus shows a complete absence of airflow, with localized re-circulation occurring around this area (Fig. 9). A reverse airflow pattern is noted in the inferior turbinate region, anterior to the airflow absence. No velocity vectors are detected in the olfactory region. Re-circulation zones are present anterior to the turbinates, beneath the olfactory region, and along the posterior boundary of the nasopharynx. The inferior meatus region exhibits high-velocity airflow, whereas the superior meatus and olfactory region show minimal airflow (Fig. 10). The superior turbinate region demonstrates reduced airflow compared to the normal nasal model.

**Fig. 11:**
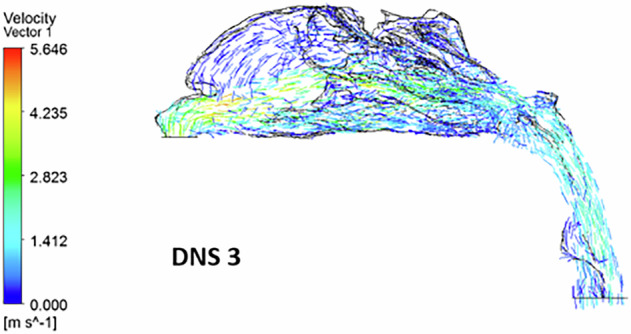
The airflow velocity vectors in a deviated nasal septum III.

**Fig. 12:**
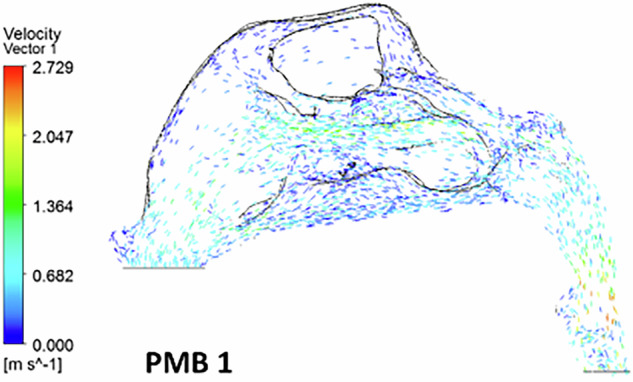
The velocity streamlines in a unilateral grade III polyp.

The velocity vector further highlights airflow re-circulation in the superior and inferior turbinate regions. Vortices are present anterior to the nasal valve, and a reverse airflow pattern is seen in the inferior turbinate region (Fig. 11). Airflow is completely absent in the superior turbinate and olfactory regions, with the flow being redirected toward the middle and inferior turbinate regions. The nasal valve region experiences diminished airflow. Additionally, localized re-circulation is observed near the floor of the superior turbinate (Fig. 12).

**Fig. 13:**
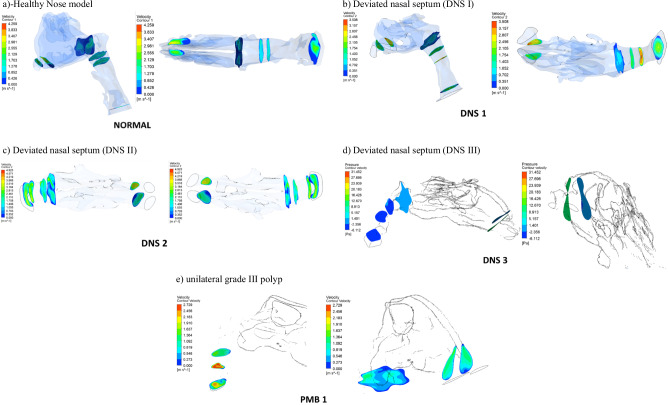
The velocity contours **a** healthy nose model, **b** deviated nasal septum I, **c** deviated nasal septum II, **d** deviated nasal septum III, **e** unilateral grade III polyp.

The velocity contour at the nostrils presents a similar pattern in individuals without nasal obstruction. However, in patients with a Deviated Nasal Septum, the velocity contour differs between nostrils, with the affected side showing a maximum velocity of 3.5 Pa and a minimum of 1.7 Pa. At the minimum cross-sectional area, the velocity gradient follows a similar pattern from the inner to the outer wall, with the highest velocity (3.5 Pa) at the lumen’s center, gradually decreasing toward the outer wall (1.4 Pa). In cases of severe obstruction, the velocity contour at both nostrils maintains a consistent gradient, but the minimum cross-sectional area appears larger than in other models. The velocity contour across all models exhibits an oval cross-sectional geometry at the nostril, nasopharynx, minimum cross-sectional area, and oropharynx (Fig. 13).

### The patterns of wall shear stress distributions within the pharyngeal airway in nasal breathers and patients with different grades of nasal obstruction

**Fig. 14:**
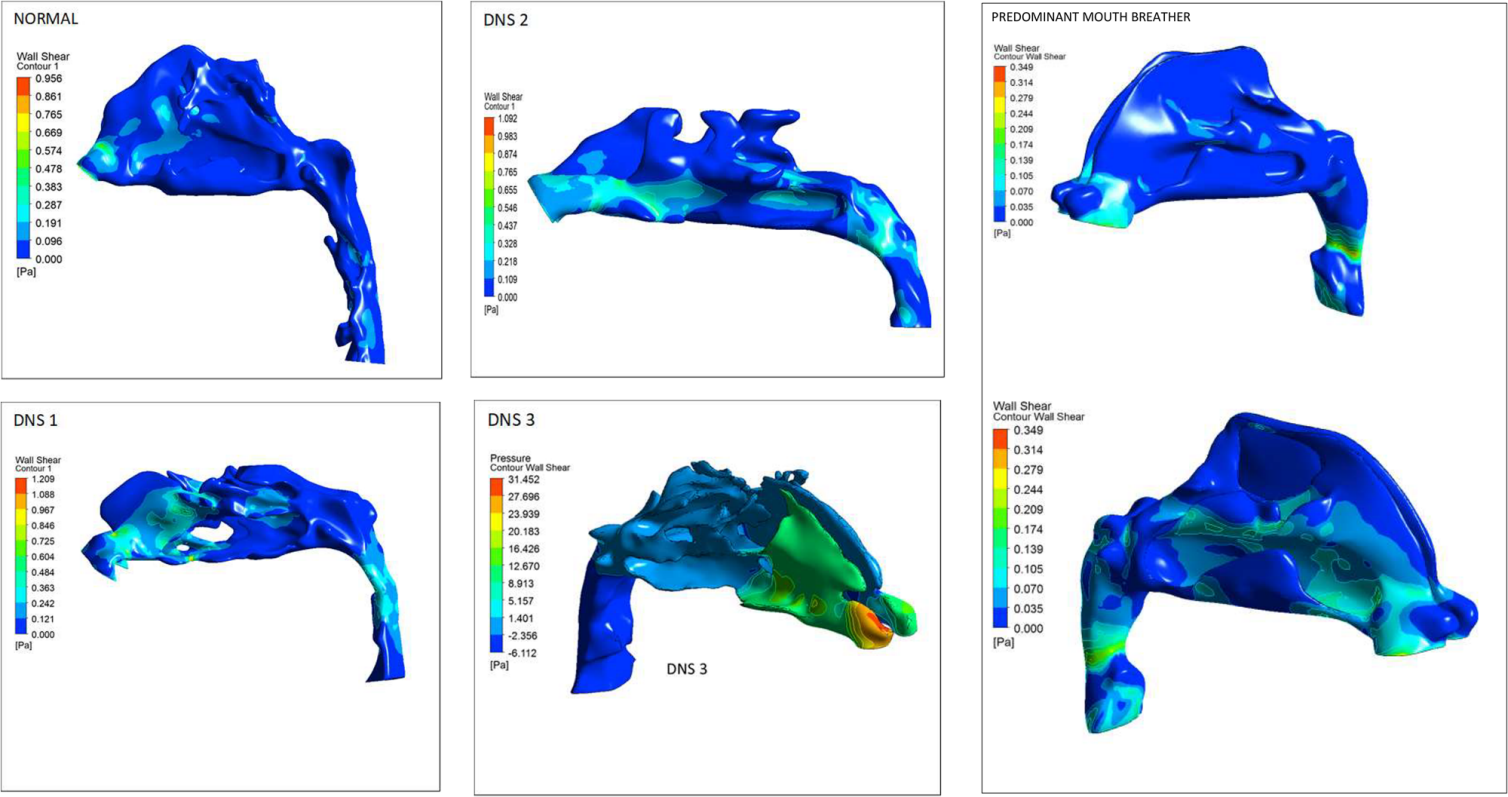
The wall shear stress in a healthy nose model.

In a normal individual, the maximum WSS ranges from 0.3 to 0.5 Pa (Fig. 14). Higher shear stress is observed in regions of airway constriction and at the minimum cross-sectional area. The inferior turbinate also experiences increased shear stress due to airflow passing through a narrow passage. Notably, the nasal valve area—encompassing the anteroinferior portion of the nasal septum and the anterior parts of the middle and inferior turbinates—exhibits elevated WSS, with maximum values ranging from 0.5 to 0.9 Pa.

Higher WSS is particularly prominent in the nostril, nasal valve area, and both the anterior and posterior sections of the middle and inferior turbinates. In the oropharyngeal region, increased shear stress is observed due to a narrower and thinner airway. In patients with a deviated nasal septum, the maximum WSS is concentrated in the nostril and nasal septum on the affected side, ranging from 20.1 to 31.4 Pa. The affected side experiences significantly greater shear stress compared to the normal side, with high values also noted in the nostril, nasal valve, and turbinate regions.

Patients with a deviated nasal septum exhibit the highest resistance to airflow, measuring 38.5 Pa. Additionally, airway volume is reduced in patients with deviated nasal septums compared to normal individuals. In cases of nasal polyps, the airway volume is measured at 3.5 m³, which is comparable to that of a normal individual (Table 1).

**Table 1:** Comparison of airway resistance and airway volume.

Case	Pressure inlet Pa	Pressure outlet Pa	Delta P, pressure Drop Pa	Air vol (m^3^)
NORMAL	16.3162 [Pa]	−0.951413 [Pa]	17.2676	3.64489e−05 [m^3^]
DNS 1	10.7939 [Pa]	−19.4585 [Pa]	30.2524	2.20795e−05 [m^3^]
DNS 2	15.1701 [Pa]	−2.69983 [Pa]	17.8699	1.35564e−05 [m^3^]
DNS 3	32.3705 [Pa]	−6.18506 [Pa]	38.5555 [Pa]	1.71807e−05 [m^3^]
Predominant MB1	7.81288 [Pa]	−5.47748 [Pa]	13.29028 [Pa]	3.56144e−05 [m^3^]

## Discussion

In summary, nasal obstruction, defined as the subjective sensation of inadequate airflow, can result from various anatomical and physiological factors. Two prevalent conditions linked to nasal obstruction are Nasal Septal Deviation, with a reported prevalence of 55%, and Nasal Polyposis, affecting 0.2–4% of the population, with a male predilection. While the precise etiology and comprehensive management of these conditions remain insufficiently documented, existing studies have investigated the effects of altered respiratory function on craniofacial growth [6]. Ackerman et al. highlighted the importance of assessing soft tissues, such as nasal polyps, in orthodontic and orthopedic treatment planning [7]. Font et al. [8] suggested a correlation between nasal obstruction and abnormal craniofacial development, while Yamada et al. [9] identified associations between nasal obstruction and specific craniofacial changes, including posterior mandibular rotation, posterior-superior condylar growth, an obtuse gonial angle, anterior open bite, and constricted dental arches.

CFD was employed to evaluate airway characteristics in individuals with normal airways and varying degrees of nasal obstruction. Using patient-specific 3D stereolithographic models derived from Computed Tomographic images, CFD enabled a non-invasive visualization of the complex three-dimensional airway while preserving natural airflow dynamics [10]. Given that age plays a role in airway development, this study focused on patients aged 22.4 ± 5 years, ensuring stable airway dimensions. The airflow characteristics were analyzed and compared among severe mouth breathers with polyposis, partial mouth breathers with septal deviation, and individuals with unobstructed airways.

Olfaction, the primary function of the nose, depends on chemical molecules carried by airflow into the olfactory epithelium. Zubair et al. [11] highlighted that posture influences the numerical conditions used in airflow simulations, while Zhao et al. [12] emphasized that flow patterns determine the volume of volatile chemicals detected by olfactory receptors, underscoring the importance of airway volume and flow rate for olfaction.

This study analyzed airflow dynamics within the pharyngeal airway. Under normal breathing conditions, airflow primarily enters the superior part of the inferior turbinate region, accounting for over half of the total airflow on one side, a phenomenon attributed to minimal resistance. Flow direction in the middle and superior meatus regions follows a parabolic pattern, consistent with findings from normal airway studies. The anatomical positioning of the middle meatus allows greater airflow at its cranial end compared to the caudal end, as the cranial end is positioned higher.

In model 1 with a deviated nasal septum, peak airflow shifts to the superior turbinate region due to redirection caused by septal deviation, leading to anosmia. In model 2, airflow is predominantly concentrated in the inferior turbinate region due to anatomical variations. The model simulating a mouth breather with chronic nasal polyposis showed localized airflow recirculation in the middle and inferior meatus regions, aligning with previous studies on nasal obstruction [13]. Reduced airflow in the olfactory region further confirmed anosmia symptoms. Pathological conditions significantly alter airflow patterns, emphasizing the impact of anatomical variations on airway dynamics. Increased airflow velocity is observed in regions of resistance, often caused by conditions such as polyposis, septal deviation, or airway constriction. This study found that higher velocities occur in areas with minimal cross-sectional area or structural abnormalities, consistent with Zhao et al. [14] findings that even minor nasal cavity changes can substantially affect airflow and odorant uptake.

Additionally, vortices, formed due to directional changes in airflow around obstructions, function as a defense mechanism, particularly in the upper nasal cavity containing the olfactory epithelium. Patients with altered nasal breathing patterns exhibit more vortices, while high-velocity regions are observed in the nasopharynx and dorsal nasal cavity in cases of moderate to severe obstructions, aligning with studies on turbinate hypertrophy [14].

In a normal patient, higher WSS was observed in the nasal valve region and the anterior portions of the inferior and middle meatus, with a bilaterally symmetrical distribution. In contrast, patients with Deviated Nasal Septum and nasal polyposis exhibited elevated WSS in the nasal septum region and lower nasopharynx on the affected side [15]. This aligns with studies showing symmetrical WSS patterns in normal breathers, whereas in deviated nasal septum cases, high WSS regions shift to the superior and inferior nasal cavities due to airflow diversion. In the model 3, increased WSS was noted in the nasal septum and affected nostril, primarily due to altered airflow resulting from the absence of superior turbinate flow. The nasal cavity’s mucosa contains receptors responsible for tactile and thermal sensations, and areas subjected to high shear stress may influence nasal secretions by triggering cellular responses in the nasal epithelium. Ramanathan et al. [16]. suggested that the intentional removal of turbinate’s, which house these receptors, reduces airflow sensation by 20%, affecting nasal air conditioning and ultimately diminishing Quality of Life.

In individuals experiencing nasal symptoms, elevated WSS is observed in the nasal valve area, as well as the anterior and posterior regions of the middle turbinate. The most constricted airway segments also exhibit increased WSS due to the narrowed lumen, leading to higher velocity and strain rates. This heightened WSS may disrupt cellular responses, impair mucociliary clearance, and contribute to a feedback loop that exacerbates airway disorders. Studies have established a direct correlation between WSS and flow rate [15], indicating that increased airflow leads to elevated WSS, which in turn can cause epithelial damage, inflammation, and airway remodeling [17].

Research by Kim et al. [18] found that nasal resistance in individuals with a deviated nasal septum is twice as high as in those with normal airways. This aligns with the findings of the present study, which also reports doubled nasal resistance in patients with a deviated nasal septum compared to normal subjects. The increased resistance is attributed to a reduced cross-sectional airway area due to obstruction, which alters directional airflow velocity. In patients with a deviated nasal septum, the pressure drop is non-uniform, with the most significant decrease occurring near the site of septal deviation or obstruction. In contrast, normal individuals exhibit a smooth and consistent pressure drop.

During inspiration, normal individuals experience a nasal pressure of −0.9 Pa, while those with deviated septum and obstruction exhibit pressures ranging from −2.6 Pa to −19.5 Pa, consistent with prior studies on moderate to severe obstruction. Patients with severe obstruction show pressures around −5.4 Pa, which aligns with the findings of Radulesco et al. [19], who reported pressure values ranging from −19 Pa to −33 Pa in cases of moderate to severe obstruction. The presence of negative pressure in nasal obstruction can impact muscle function, reducing upper airway muscle caliber and potentially increasing the risk of snoring and hypopneas.

In normal breathing, bilateral nostril velocities are comparable. However, in patients with a deviated nasal septum, velocity is higher on the affected side due to altered airflow dynamics. In cases of severe obstruction, the velocity at the minimum cross-sectional area is reduced, likely influenced by variations in pharyngeal airway anatomy. Airflow distribution remains relatively even across the cross-sectional area, with lower velocity near the outer wall and higher velocity concentrated at the lumen center.

A limitation of our study was the relatively small sample size used for the CFD-based evaluation of airflow dynamics, which may affect the generalizability of the findings. To strengthen future research, similar studies should be conducted with a larger, better-matched sample to enhance the reliability and generalizability of the findings. Additionally, further investigation into airway characteristics and airflow variations between normal, unobstructed airways and those with varying degrees of anatomical nasal obstructions, particularly focusing on their long-term impact, would offer valuable insights into the progression and clinical consequences of impaired nasal breathing.

## Conclusion

A comparative analysis of airway characteristics and airflow variations between a normal, unobstructed airway and varying degrees of anatomical nasal obstructions was conducted using CFD. This patient-specific simulation approach enabled a detailed assessment of airflow behavior within the upper airway under different physiological and pathological conditions. Our study revealed several notable findings regarding pharyngeal airflow. First, airflow patterns demonstrated a non-uniform velocity distribution, largely influenced by the anatomical complexity of the nasal cavity. Significant variations in airflow velocity were observed not only between individuals but also within different regions of the same nasal compartment, highlighting the dynamic nature of airflow in response to localized structural differences. In cases of nasal obstruction, particularly those involving polyposis or septal deviation, there was a marked reduction in airflow through the olfactory region, which correlated with the clinical symptom of anosmia. Furthermore, pressure drop along the airway was inconsistent in individuals with nasal obstruction, with more substantial drops occurring near the obstructed segments. These localized pressure changes may contribute to altered breathing mechanics and compensatory mouth breathing. The airflow characteristics documented in this study offer valuable insights into the interplay between normal and impaired nasal respiration, emphasizing the significance of anatomical obstructions in influencing flow dynamics. By comparing airflow patterns between nasal and mouth breathers, the analysis allows for the identification of critical regions of flow disruption and the quantification of functional impairment. These findings not only enhance our understanding of airway physiology but also provide a practical framework for clinicians to evaluate the severity of obstruction and develop targeted, comprehensive treatment strategies tailored to individual patient needs.

## Data Availability

Any other information about this article can be made available on reasonable request via an email to the corresponding author.
