# Correction: Understanding airflow dynamics: a computational study of nasal and oral breathers using patient-specific models

**DOI:** 10.1038/s41405-026-00447-8

**Published:** 2026-05-22

**Authors:** Shawn Ann Thomas, Supriya Nambiar, Mohammed Zuber, S M Abdul Khader, Athira V

**Affiliations:** 1 Department of Orthodontics and Dentofacial Orthopaedics, Manipal College of Dental Sciences Mangalore, Manipal Academy of Higher Education, Manipal, Karnataka 576104 India; 2 Manipal Institute of Technology, Manipal Academy of Higher Education, Manipal, Karnataka 576104 India

**Keywords:** Craniofacial orthodontics, Cone-beam computed tomography

Correction to: *BDJ Open* 10.1038/s41405-025-00357-1, published online 15 January 2026

In this article the author’s name S M Abdul Khader was incorrectly written as Abdul Khader. The original article has been updated.

